# Upon the Use of Opium in Certain Conditions of the Porturient Process*Read before the Leavenworth Medical and Surgical Association.

**Published:** 1859-06

**Authors:** C. A. Logan

**Affiliations:** Leavenworth City, Kansas


*Upon the Use of Opium in Certain Conditions of the Porturient Process.** By C. A. LOGAN, M. D., Leavenworth City, Kansas.

Foremost amongst the many difficulties that the accoucher is obliged to encounter in his daily experiences stand the derangements of the

*Read before the Leavenworth Medical and Surgical Association.

propulsive and expulsive powers of the uterus itself. Distortions of the pelvis and malpresentations of the child, in their various forms, constitute, it is true, obstacles which occasionally render the process of parturition one of serious and portentous import; yet these, in a very large majority of cases, are overcome by a scientific knowledge of the relations and adaptations of the fœtus to the pelvic cavity, with safety to both mother and child. But the motor forces, which launch the young being into the world, are resident within the maternal economy, and consequently, being less under our control, frequently become the most vexatious and unmanageable impediment to a safe and speedy delivery. Our advance in this department of labor has in no wise been commensurate with that of the mechanism of the process, and consequently when the world was presented with a remedy that was thought to possess a controlling influence over the uterine fibre, it was greedily seized upon and applied indiscriminately to every case in which the uterine forces were inefficient; and with what result thousands of childless mothers can tell. That ergot is a powerful agent, for good or evil, no one will deny; but that it is utterly inadmissible in a very great number of cases of uterine inefficiency every experienced practioner will bear testimony.

My object in writing this paper is to narrate briefly a form of treatment I have been adopting for the last few years, in a variety of tedious labor with which probably we are all familiar, which has been attended with very great success in my hands.

We are summoned, hastily, to see our patient, whom we find has been in labor, perhaps, some hours, with the most excrutiating pains, and, upon inquiry, we find that the pains are confined almost wholly to the back; there is little or no effort at "bearing down," but almost a continual outcry, for the pains scarcely leave at all. A vaginal examination reveals to us the fact, that notwithstanding the severity of the pains, the membranes scarcely become tense; there is, in fact, no propulsive action exerted upon the ovum which occasions it to advance one inch. The parts may be soft and perfectly natural; the os may be dilated to any extent compatible with the integrity of the membranes; and when dilated we generally find that the pains came on naturally enough at first, but after an uncertain period lost their expulsive character and assumed that which we are now considering.

These pains are of the most exhausting nature to the woman, and frequently, in her anguish, she will cry out, "Oh, Doctor! I shall die; I know I shall!" And, upon my word, in my early experiences, I have really thought she would. Now, this species of pain, although frequently met with in females of a robust and plethoric habit, is much more likely to happen to those of a delicate and nervous conformation.

I have remarked that this irregular kind of pain may come on at any time prior to the expulsion of the child, and not, as asserted by Madame Boivin, only before the head has passed the superior strait. I have known it occur after the head has escaped from the uterus.—Here, however, the pain and impediment to delivery is produced by a somewhat different state of things from that which occurs before the rupture of the membranes. In the former case, the neck being subjected no longer to the mechanical dilatation of the child's head by the same species of irregular contraction as occurs in the last form, becomes constricted around the neck of the child, thus preventing the shoulders from passing even, were the *vis a tergo* of a natural character.

This kind of pain has been commented upon by more than one observer. Dr. Dewees, in his excellent treatise on midwifery, in speaking of its cause, remarks, that "it has been attributed by some to the stretching of the posterior ligaments of the uterus; by others, to the violent contraction of the muscles of the posterior part of the trunk.—We are of the opinion it is caused by *some irregular action of the uterus itself.*"

In contemplating the dynamical forces of parturition, it is not necessary for our present purpose to go into an investigation of their final and efficient cause: whether it is due to the inherent contractility of the uterus, as maintained by many, or as the result of a "reflex" action of the spinal cord, as elucidated by Dr. Tyler Smith, concerns us at present. We have but to examine the structure of the uterus, to find it composed of muscular fibres disposed in such a way that their united and harmonious action has the effect of diminishing its cavity in all directions. In order, then, that the action of the uterus should be efficiently exerted upon its contents, it is necessary that an equable and steady balance must exist between the longitudinal and circular fibres (I do not refer to the semi-circular fibres disposed around the cervix; and if, through a perverted nervous action, or an exhausted condition of the muscular fibre, or any other cause, the unity of action should be destroyed between the two sets of fibres, it is evident that the object will be defeated so long as this disproportion continues. Now, this is what I conceive to take place in the kind of cases I have referred to. The uterus, being muscular in its character, must be subject to the same laws that govern the muscular tissue generally; and hence we find that in females of a delicate habit, in whom the nervous energy is deficient or unequal, this state of things is apt to exist; or in those in whom from my cause—as a large head, a large quantity of liquor amnii, etc.,—the dilatation has been tedious, and the muscular structure has been subjected to long continued and violent action, this spasmodic condition is apt to obtain; also at the commencement of labor, and especially in primiparous cases, before the simultaneousness of the movement is developed, it presents itself; and, finally, it is seen frequently in those women of a full and plethoric habit, in whom an undue supply of blood predisposes to a morbid irritability and contractility of the muscular tissue generally. In the latter cases, the lancet freely applied constitutes a weapon of never failing efficacy.—It is in the first mentioned conditions that I wish to present *opium* as a remedy of untold potency for arresting the irregular action of the uterus, and restoring the pains, paradoxical as it may seem, to their original and proper condition; and this it does by simply equalizing the perverted nervous action, allowing, nay, compeling a uniformity of action in the uterine fibres. Nor does it have the effect, in a large majority of cases, as might be supposed, of putting an end to all pain: on the contrary, I have seen women shrieking almost incessantly with this form of pain in the back without any expulsive action of the uterus, under the repeated administration of an opiate gradually become calm, have a decided interval established between the pains, and themselves gradually merged into powerfully expulsive efforts, which would terminate more rapidly than I dared hope in the birth of the child.—I have, at times, been astonished at the magical change, and never more than the first time I had occasion to prescribe the remedy. The woman was in the condition before described; an examination showed the os to be dilated to about double the size of a dollar, but there was no advance of the child whatever; and thinking to quiet the pains, in order that the uterus might obtain a period of rest, I prescribed an opiate, to be given every twenty or thirty minutes until the *pains were stopped*, and, promising to call again, I took my leave. I had not been gone long, however, before a messenger requested my immediate attendance. My surprise may be imagined, upon arriving, to find the child born. I was informed that she had taken two doses of the opium, and that after taking the first the old pains began to leave and become bearing down; and that after taking the second they became very violent; and in three-quarters of an hour after the first dose the child was born. An old lady, who had been her attendant, confidentially said to me, "Doctor, when you give such *powerful stuff* to bring it on, you ought to stay by." I took the hint, and have used it to advantage ever since.

Ergot, it will be perceived, in this case would have been inadmissible at the time of her taking the opium, for I believe it is an established rule never to give ergot unless the os is not only dilatable, but fully dilated. Nor do I believe that bleeding, that mighty weapon so heroically used by some in the parturient condition, would have been attended with the success of the opium ; and I am sure the patient was far better off, and convalesced much more speedily, than had a profuse bleeding been practiced. Say what we will, there can be no doubt that the excessive loss of blood to which many women are subjected has been a most fruitful source of the diseases and mortality incident to the puerperal state. No man can estimate its efficiency more highly than myself, yet, like ergot, it is not a specific for *all* the ills of parturition ; and it is only when based upon a rational consideration of the conditions present that its advantages, stripped of its deleteriousness, may be realized. I have seen women rise from child-bed so blanched, so totally bloodless, as more nearly to resemble an emaciated wax figure than anything else. I mention these things to illustrate the unpardonable abuse of remedies that so many of us are liable to be guilty of.

In this connection, I may be permitted to extract one of the many cases from the pages of my note book : "September 13, 1856. Mrs. John W———, taken to bed with her fourth child. Has always had an excessive, lingering labor. Was taken with pain early in the morning, but being aware of the length of time she always suffered, did not send for me until midnight. Arrived, I found her in intense agony, complaining bitterly of her back, while the pain never left her. Pulse weak, skin cool, no vomiting. An examination revealed the os largely dilated, but there was little or no advance of the head during the pains. Parts soft and yielding. She tells me that this has been the character of her previous labors, and that always before she had been largely bled, but which seemed to do her no good. I gave her thirty drops of tinctura opii every twenty or thirty minutes, and soon the pains began to leave the back, and, becoming violently expulsive, the child was born in one hour and a quarter. She declares it to be the 'quickest' time she ever had. Convalescence rapid."

This case is illustrative not only of the efficiency of opium in certain cases, but also of the misapplication of blood-letting in her previous labors. Even in cases where the requisite conditions for the administration of ergot is present, I prefer the opium, if it is adapted to the case: first, because I regard it as eminently more certain; and second, because I do not believe it is accompanied with any of the perils of ergot. I speak now of its judicious and proper administration: all remedies are liable to abuse, as I have before mentioned.

I generally use the sulphate of morphia, in a simple aqueous or aromatic solution, in doses varying from the one-eighth, one-fourth, to even one-half of a grain, according to the circumstances, repeated every twenty or thirty minutes until the pains either change their character or cease altogether, which latter condition is greatly more favorable than the distressing pains, which do not advance the labor at all, but on the contrary, exhaust and prostrate the patient to the last degree. Besides, after a period of rest, the uterus generally is aroused to a healthy and vigorous action, which soon completes the labor. I have never seen any untoward symptom arise after its administration that could fairly be attributed to its use; and, when given in the gradual and cautious manner I have indicated, not even an unusual amount of drowsiness to supervene. Neither have I seen a case of hemorrhage follow it.

The frequent and indiscriminate use of ergot by injudicious practitioners has come to be a crying evil. It is not that we do not possess the remedies to combat these cases of lingering labor dependent upon a deficiency of uterine power, that we are obliged to wait for hour after hour with vexatious impatience, exhausting not only the woman but ourselves; but it is rather because we do not inquire strictly into the pathological conditions present, and apply our remedies knowingly, but give ergot, perhaps, when we ought to bleed; bleed when we ought to give ergot; or do both when a few doses of opium would produce a happy termination for all parties.

Specifics in labor, like specifics in disease, are the consequence of ignorance, and attended with the same disastrous results. If one-half the elaborate investigation had been applied to the study of the motor forces of parturition, normally and abnormally considered, together with their dependencies generally, that has been bestowed upon the elucidation of their primary exciting cause, the long catalogue of lingering labors would be very materially diminished, and a real benefit conferred upon suffering humanity.—*Cincinnati Lancet & Observer.*

